# Comparative analysis of reproductive tract microbiomes in modern and slower-growing broiler breeder lines

**DOI:** 10.3389/fvets.2024.1386410

**Published:** 2024-04-10

**Authors:** Naama Shterzer, Yara Sbehat, Binita Poudel, Nir Rothschild, Olanrewaju Eunice Oloko, Shelly Druyan, Erez Mills

**Affiliations:** ^1^Department of Animal Sciences, Robert H. Smith Faculty of Agriculture, Food, and Environment, The Hebrew University of Jerusalem, Rehovot, Israel; ^2^Department of Poultry and Aquaculture Science, Agricultural Research Organization, Volcani Center, Rishon LeTsiyon, Israel

**Keywords:** broiler breeding, hen reproductive tract microbiome, growth targeted selection, genetics, reproductive physiology

## Abstract

**Introduction:**

The reproductive tract microbiome in hens is of interest because bacteria in the reproductive tract could potentially affect fertilization and egg production, as well as integrate into the forming egg and vertically transmit to progeny.

**Methods:**

The reproductive tract microbiome of 37-week-old modern commercial Cobb breeding dams was compared with that of dams from a broiler Legacy line which has not undergone selection since 1986. All animals were kept together under the same management protocol from day of hatch to avoid confounders.

**Results:**

In regards to reproductive abilities, Cobb dams’ eggs weighed more and the magnum section of their reproductive tract was longer. In regards to microbiome composition, it was found that the reproductive tract microbiomes of the two lines had a lot in common but also that the two breeds have unique reproductive tract microbiomes. Specifically, the order Pseudomonadales was higher in the magnum of Legacy dams, while Verrucomicrobiales was lower. In the infundibulum, Lactobacillales were higher in the Legacy dams while Verrucomicrobiales, Bacteroidales, RF32 and YS2 were lower.

**Discussion:**

our results show that breeding programs have modified not only the physiology of the reproductive tract but also the reproductive tract microbiome. Additional research is required to understand the implications of these changes in the reproductive tract microbiome on the chicken host.

## Introduction

While the gut microbiome is the focus of intense study, the hen’s reproductive tract microbiome has only come into focus recently. The reproductive tract consists of the vagina, shell gland (uterus), isthmus, magnum, and infundibulum. The infundibulum is the site of fertilization. From the infundibulum, the egg passes to the magnum, which is the longest section of the oviduct and where the first layers of albumen are added. Next, the egg moves to the isthmus, in which the inner membrane and outer shell membranes are added. The outer shell and pigments are added in the uterus. Finally, the ready egg exits through the vagina and cloaca to the outer environment. It is important to note that gut content also empties into the cloaca before exiting to the external environment.

Basic characterization of the reproductive tract microbiome showed that this microbiome has higher richness than that of the small intestine, but lower than that of the cecum (1, 2). A comparison between different segments of the reproductive tract shows that while the vagina microbiome is unique, the other segments do not differ significantly (1–4). A large overlap of bacterial species between the gut and reproductive microbiomes has been shown, as well as a correlation between the relative abundance of bacterial strains in the gut and the chance they will be identified in the reproductive tract, implying the gut microbiota is an important source for the reproductive tract community (1). Finally, it was shown that richness in the reproductive tract increases upon sexual maturity (3).

A correlation between egg laying and the reproductive tract microbiome was identified (2). *Lactobacillus*, *Bacteroides*, and *Desulfovibrio* found in the reproductive tract were positively correlated with egg production, while *Pseudomonas*, *Exiguobacterium*, and unidentified Erysipelotrichaceae were negatively associated. Another study looking at interactions between reproductive tract bacteria and egg production phenotypes identified a pair of bacterial genera, *Staphylococcus* and *Ralstonia*, associated with darker eggshells, and a genus, *Romboutsia*, negatively associated with egg number (4). It should be noted that these studies cannot determine the causality of these associations. In humans, different compositions of the reproductive tract microbiota are associated with infection and inflammation (5) and are possibly linked to reproduction ability and infertility (6). To conclude, it is possible that the reproductive tract microbiota affects fertilization and egg formation.

Another possible role for the reproductive tract microbiome is to enable the vertical transmission of hen commensal microbes to chicks. This is because any bacteria found in the reproductive tract are likely to find their way into the egg white or the eggshell. Indeed, a comparison of the maternal magnum and cloaca, descendent egg shell, egg white, and embryo gut identified 21 core genera (3), implying vertical transmission through the reproductive tract was occurring. Furthermore, as mentioned earlier, because the gut is likely a major source of reproductive tract bacteria (1), it is possible that the reproductive tract is a part of the mechanism for vertically transmitting gut bacteria. It is important to note that to vertically transmit reproductive tract bacteria, these bacteria must also survive in these environments until they can colonize chicks. A study comparing the fecal microbiomes of hens and their chicks found that while some bacterial strains were shared between hens and chicks and were possibly utilizing a reproductive tract route for vertical transmission, most of the fecal bacterial strains found in hen fecal samples were not found in progeny in commercial-like conditions in which chicks were hatched and grown in the absence of adults (7). However, the few bacterial strains that were shared between hens and chicks and were possibly vertically transmitting, were found both in the magnum of breeders as well as on eggshells, and were the most abundant and prevalent strains in the chicks. Finally, in an analysis of possible introduction methods for the probiotic strain *Enterococcus faecium* it was shown that broiler breeders drinking water supplemented with *Enterococcus faecium* could vertically transmit this probiotic strain to progeny (8). Though the exact route of transmission was not characterized, it can be speculated to include the egg and possibly the reproductive tract. To conclude, the reproductive tract microbiota may affect the development of the gut microbiome of the next generation, including the possible transmission of probiotic bacteria.

Due to the possible roles of the reproductive tract microbiota, it is also important to understand if genetics, and more specifically the genetic effect of breeding, has an impact on this microbiota. A previous study looking into the association of host genetics with the reproductive tract microbiota and the gut microbiota of 128 layer chickens found only a weak association (2). Furthermore, it was also shown that genetic kinship had no effect on reproductive tract microbiome composition (4). It is important to note both studies used genetic variation within pure lines, and therefore any associations are likely to be limited compared to the genetic differences between different breeds. A number of studies have shown that breeding modulates the gut microbiota (9–14). Because the intestinal and reproductive tract microbiotas are connected, it is likely that breeding also modulates the reproductive tract microbiota. In this regard, we have previously compared the cecum microbiome of 37-week-old commercial Cobb breeding dams with dams from a broiler Legacy line which has not undergone selection since 1986 (13). This comparison showed that the two microbiomes were different. A major difference was the presence of genus *Akkermansia* as a major community member in modern Cobb but not in Legacy hens. Considering that the gut microbiome is a source of reproductive tract bacteria we hypothesized that the reproductive tract microbiomes of these birds were also different. Here we analyzed the reproductive tract microbiomes of these same Cobb and Legacy hens as well as some of their reproductive and physiological parameters.

## Materials and methods

### Genetic lines

All animal trials were subjected to approval by the Hebrew University of Jerusalem’s Ethics committee, approval No. AG-19-15897-3, and were conducted in accordance with the guidelines of the National Council for Animal Experimentation.

Two genetic lines were sampled: Cobb – the current modern breeder line; and Legacy – a local Israeli broiler line which has not been under selection pressure since 1986 (13, 15, 16).

### Growth conditions and elimination of confounders

Eggs from both lines were incubated and hatched on-site. A total of 62 Cobb breeders and 84 Legacy breeders were housed in the same chicken house, under identical conditions, and managed by the same personnel from hatch and throughout the entire experiment. At six weeks of age, the birds were individually placed in cages measuring 45×45 cm. All birds were raised following the same management protocol (Cobb Breeder Management Guide, 2018), which included uniform feeding practices (for feed nutritional composition see Supplementary Table S1).

During the production stage, birds were fed once a day in the morning, adhering to the feeding tables outlined in the management protocol. At 24 weeks of age, all birds were moved to cages in an open shed and exposed to 16 h of light per day. Eggs were manually collected twice daily, and individual laying was monitored. At 37 weeks of age, when both lines had reached their peak laying state and were still producing at high levels, the birds were dissected, and internal samples were collected. This was done regardless of the egg production cycle stage they were in.

It is assumed that by waiting until the age of 37 weeks, there was sufficient time for microbial transfer between animals to compensate for any differences in initial exposure. Additionally, selecting this time point ensured that differences observed between the birds were not merely the result of variations in effective physiological age.

### Sample collection

Ten animals of each line were randomly selected at age 37 weeks and euthanized by cervical dislocation. The infundibulum was removed and placed into sterile PBS, and the magnum mucosa was scraped with a sterile glass slide into sterile PBS. All samples were flash frozen in liquid nitrogen and kept at −20°C until DNA extraction.

### DNA extraction

700 μL of magnum samples or the entire infundibulum samples were mixed with 700 μL of Tris-saturated phenol and 100 μL of 10% SDS. The mixture was disrupted with 0.1 mm glass beads following phenol-chloroform extraction, as described by Stevenson & Weimer (17). DNA was subsequently precipitated with isopropanol and suspended in DDW.

### 16S rRNA gene sequencing

The 16S rRNA gene library underwent preparation and sequencing following the Earth Microbiome Project protocol (18) with V4 primers 515F (GTGYCAGCMGCCGCGGTAA) and 806R (GGACTACNVGGGTWTCTAAT). 250 bp paired-end sequencing was conducted on an Illumina Miseq platform using a V2 reagent kit by Hylabs (Rehovot, Israel), and yielded 16,820 ± 9,073 reads per sample. One sample failed to be sequenced. QIIME2 version 2020.11.1 (19) was utilized for sequence processing and taxonomy assignment. Dada2 plugin version 2020.11.1 (20) using the denoise-paired method was used to determine amplicon sequence variants (ASVs). Truncation of all reads at position 200 was applied. After quality control, 11,573 ± 7,192 reads per sample remained. ASVs with less than 5 reads in the whole dataset were excluded, and all samples were normalized to 4,000 reads per sample. A total of 1,660 ASVs were observed. Taxonomy was assigned using a naive-bayes classifier (21) trained on the Greengenes database (22). ASVs with the taxonomic assignment of “Bacteria” were compared to the NT database using BLAST (23) and those 100% identical to *Gallus gallus* mitochondrion were removed.

### Statistical analysis

All statistical tests were done using GraphPad Prism 8.0.0 (GraphPad Software, San Diego California USA^1^), except ANOSIM tests which were performed using Past 4.05 (24).

## Results

### Differences in reproductive tract physiology and egg production parameters between Cobb and Legacy breeders

**Figure 1:**
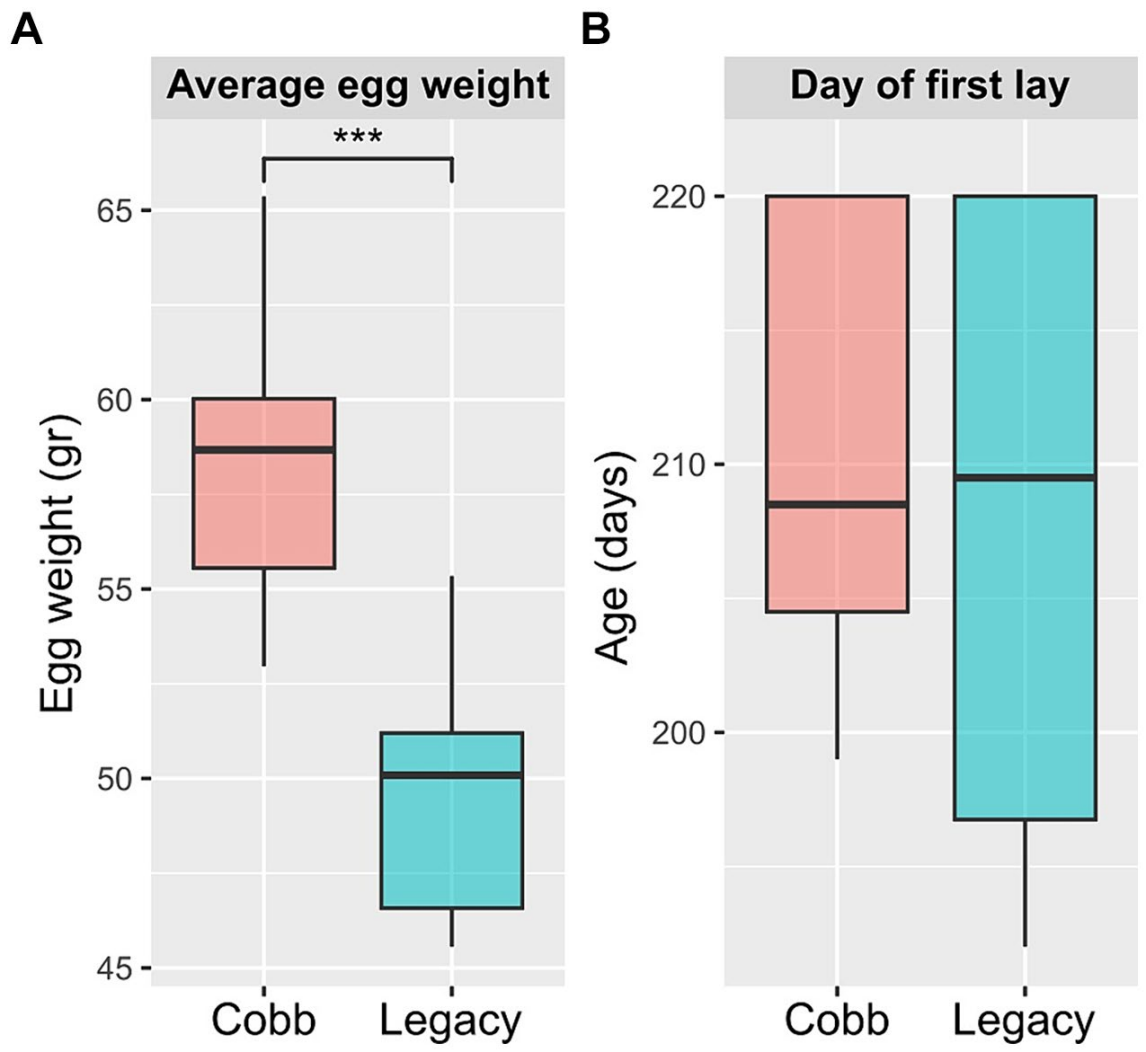
Egg production parameters of Cobb and Legacy dams. **(A)** Average egg weight. **(B)** Day of first egg lay. ***Mann–Whitney *p* < 0.001.

To determine if breeding programs aiming for faster growth and meat yield had an effect on egg production, egg laying and egg weight for the two breeds were analyzed. An analysis of egg weight showed Cobb eggs were heavier than Legacy breeders (Figure 1A). Furthermore, Legacy breeders began laying eggs 2.7 days before Cobb breeders but this difference was not statistically significant (Figure 1B).

**Figure 2:**
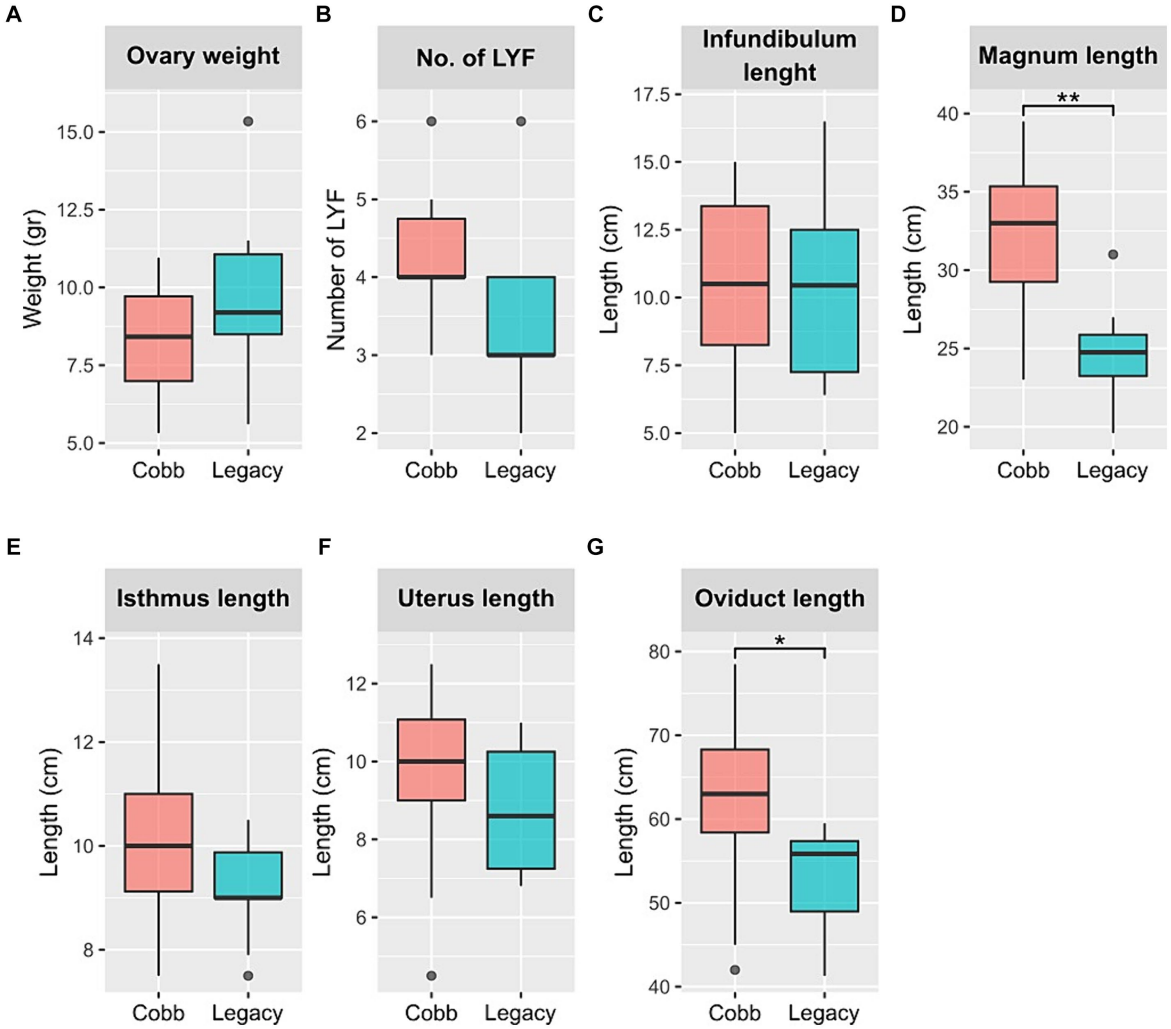
Physiological parameters of Cobb and Legacy dams. **(A)** Ovary weight. **(B)** Number of large yellow follicles (LYF). **(C)** Infundibulum length. **(D)** Magnum length. **(E)** Isthmus length. **(F)** Uterus length. **(G)** Total oviduct length. *Mann–Whitney *p* < 0.05, **p < 0.01.

Next, physiological reproductive parameters were compared. These included ovary weight and the number of large yellow follicles (LYF), as well as the lengths of the different sections of the reproductive tract. The number of LYF and ovary weight were not different between the two lines. Likewise, the lengths of the infundibulum, isthmus, and uterus were also not different. However, the magnum was longer in Cobb dams, and as a result the overall oviduct length was also longer in Cobb dams (Figure 2). The findings concerning egg white and magnum length implied that the reproductive tract environment has changed following breeding and therefore the resident microbiome might have also changed.

### The reproductive tract microbiome of the two lines is different

**Figure 3:**
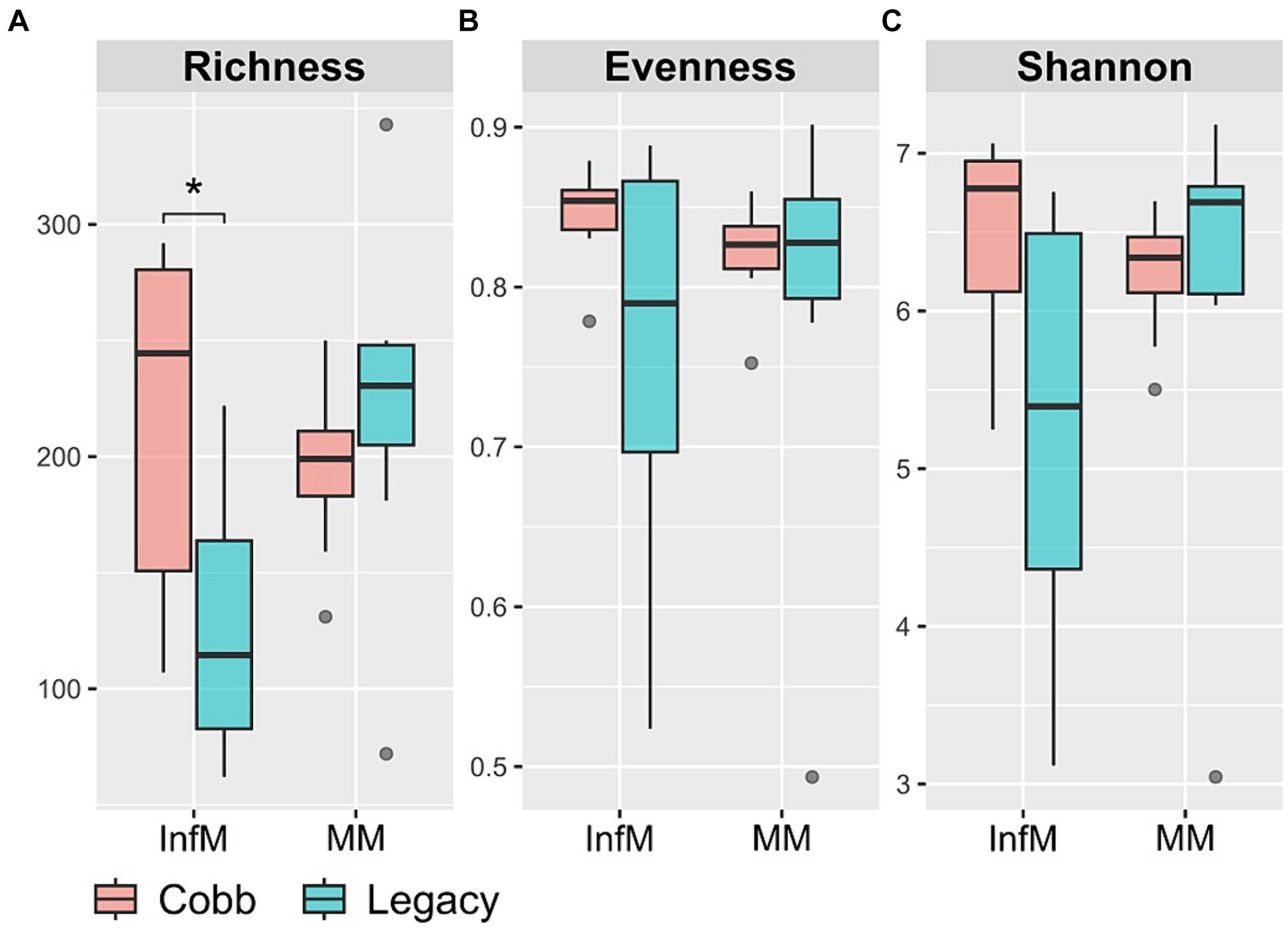
Alpha diversity measures in infundibulum (InfM) and magnum (MM) samples. **(A)** Observed ASVs. **(B)** Evenness. **(C)** Shannon diversity. *Mann–Whitney *p* < 0.05.

We chose to focus on two parts of the reproductive tract, the magnum and the infundibulum, as the magnum is the longest part of the chicken reproductive tract and is characterized by secretions of many antimicrobials, and the infundibulum is the place in which fertilization takes place. As a first step to characterizing the reproductive tract microbiome, richness, evenness, and Shannon index were analyzed. While diversity indexes of the magnum microbiome were similar between the two lines (Figure 3), the infundibulum microbiome of Legacy dams had a statistically significant lower richness.

**Figure 4:**
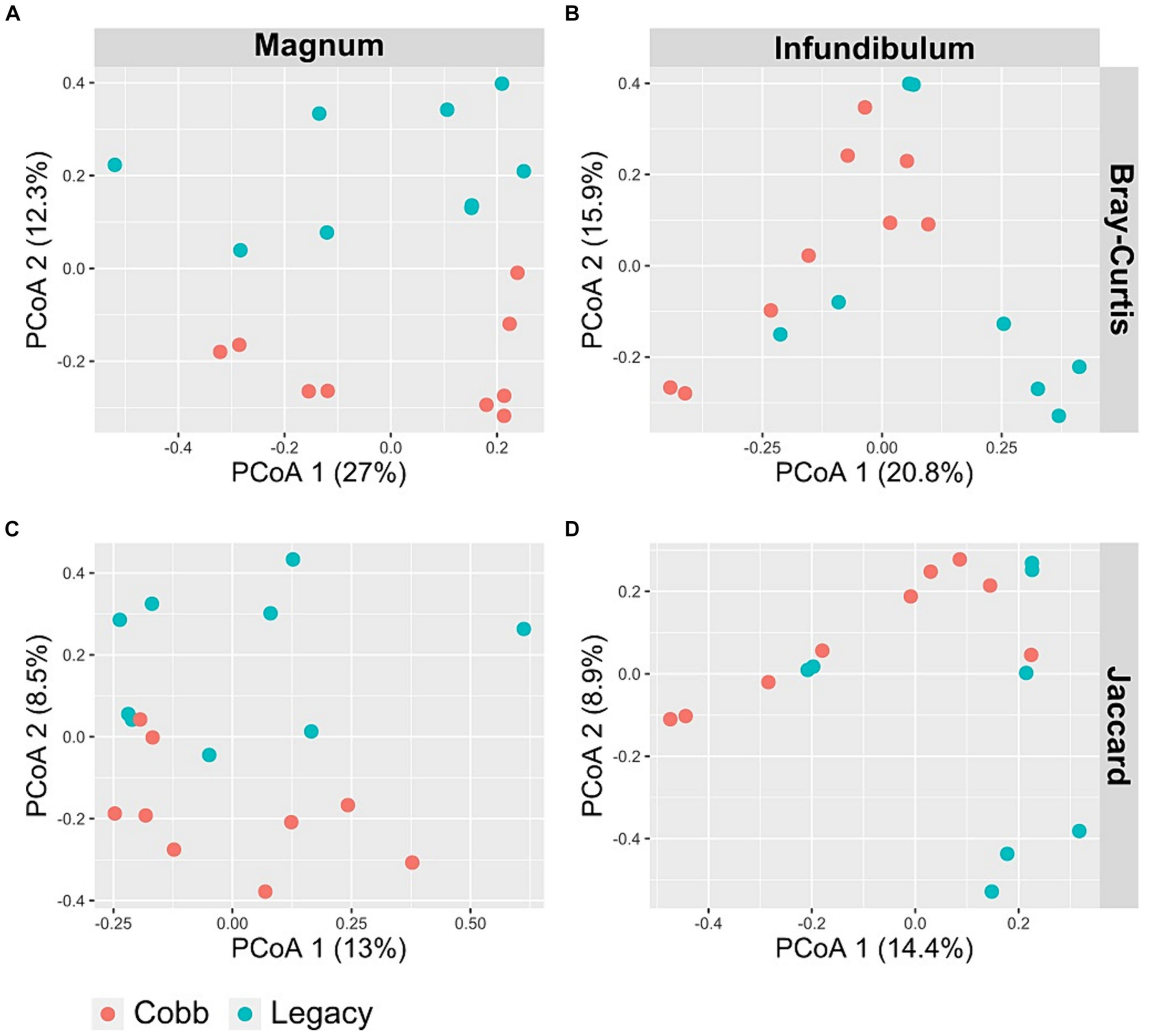
PCoA of magnum **(A, C)** and infundibulum **(B, D)** samples using Bray-Curtis **(A, B)** and Jaccard **(C, D)** indices.

To determine if the composition of the reproductive tract microbiome was different between the two lines, two dissimilarity indexes were analyzed, Jaccard and Bray-Curtis. Analysis of similarity using both indexes showed a significant difference in the magnum microbiomes of Cobb and Legacy hens (ANOSIM *p* = 0.0204 and *p* = 0.0185 for Bray-Curtis and Jaccard, respectively; Figures 4A,B). A similar analysis of the infundibulum microbiome identified a statistically significant difference between Cobb and Legacy hens using Jaccard (ANOSIM *p* = 0.029, Figure 4C) and only a trend for Bray-Curtis (ANOSIM *p* = 0.0647, Figure 4D). As Jaccard index is based exclusively on the presence and absence of ASVs, while Bray-Curtis also integrates relative abundance data, this implies that differences between the oviduct communities of Cobb and Legacy dams are based on both the ability of specific strains to colonize the different lines as well as their ability to grow to large numbers and perhaps compete with other members of the microbial community. It should be noted that the R values ranged from 0.2191 to 0.1241 showing that while the microbiomes of the two lines differed, they also had a lot in common.

### Differentially abundant bacterial orders and genera

**Figure 5:**
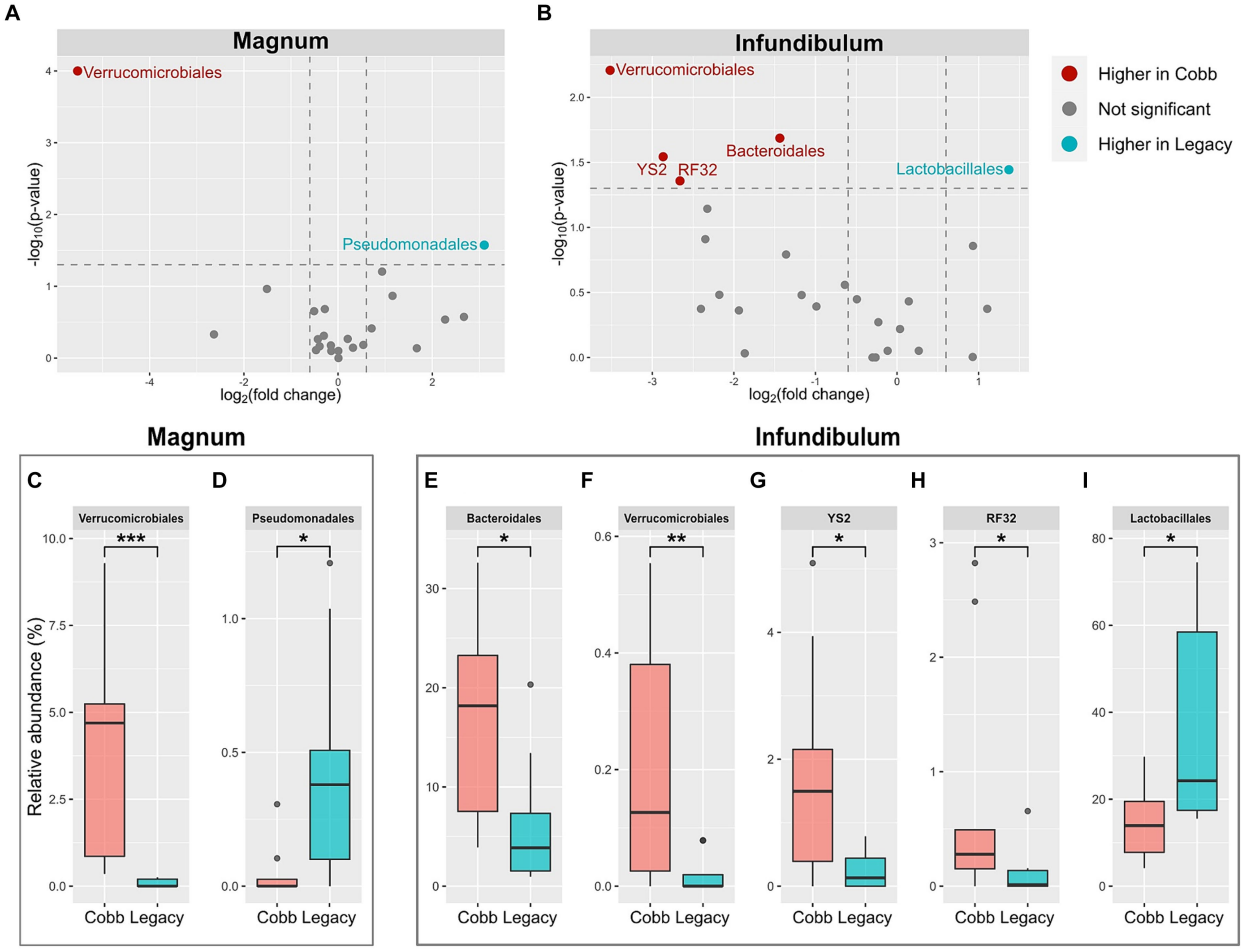
Differentially abundant orders between Cobb and Legacy dams in magnum and infundibulum samples. **(A,B)** Volcano plot of orders with average relative abundance >0.1% in the magnum **(A)** and infundibulum **(B)**. Orders with relative abundance significantly higher in Cobb dams are marked red, orders with relative abundance significantly higher in Legacy dams are marked blue, and orders not significantly different are marked gray. **(C-I)** Relative abundance of orders significantly different between Cobb and Legacy dams in the magnum **(C, D)** and infundibulum **(E-I)**. *Mann–Whitney *p* < 0.05, **p < 0.01, ***p < 0.001.

Because the microbiomes of the two breeds were found to differ, a differential abundance analysis was used to identify specific orders and genera that were different between the two breeds (Figures 5A,B; Supplementary Figures S1A,B). This analysis identified two orders which were different between Cobb and Legacy dams in the magnum, Verrucomicrobiales, and Pseudomonadales (Figures 5C,D), as well as seven genera (Supplementary Figures S1C–I). An analysis for the infundibulum samples found that Verrucomicrobiales was also different between Cobb and Legacy dams in this section of the reproductive tract, alongside Lactobacillales, Bacteroidales, YS2 and RF32 (Figures 5E-I). Five genera, representing these orders, were also found to be different in the infundibulum (Supplementary Figures S1J–N). In the magnum, Verrucomicrobiales was higher in Cobb dams, while Pseudomonadales was higher in Legacy dams. Verrucomicrobiales was also higher in Cobb dams in the infundibulum, alongside Bacteroidales, YS2 and RF32, while Lactobacillales was higher in Legacy dams. Importantly, most of these orders had relative abundances of more than 1%, and thus these differences were of important components of these microbiomes. To conclude, the reproductive tract microbiomes of Cobb and Legacy hens were characterized by differentially abundant orders and genera.

### Associations between physiological parameters and deferentially abundant orders and genera

**Figure 6:**
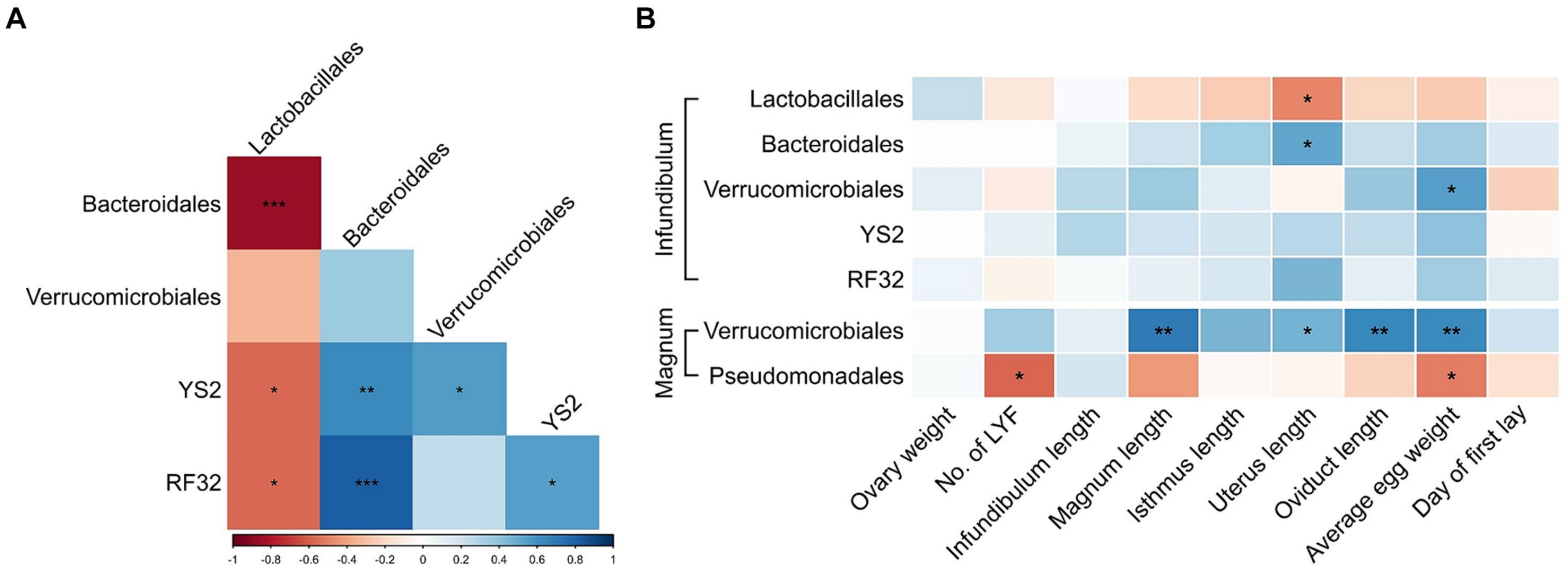
Associations between differentially abundant orders and physiological parameters. **(A)** Spearman correlations between differentially abundant orders in the infundibulum. **(B)** Spearman correlations between differentially abundant orders and physiological parameters in the infundibulum and magnum. Spearman r values are represented by a color scale, and correlation significance is denoted with asterisks. *p < 0.05, **p < 0.01, ***p < 0.001.

Because a previous analysis of cecum bacteria of these same birds showed associations of some of these orders (13) we tested whether the same associations can be identified in the reproductive tract microbiome. In the infundibulum, we found that the relative abundances of most of the differentially abundant orders were correlated (Figure 6A). In the magnum, Verrucomicrobiales and Pseudomonadales levels were also negatively correlated (Spearman *p* = 0.0118, r = −0.5791). Additional correlations were observed between the differentially abundant genera (Supplementary Figure S2).

Furthermore, correlations were also observed between differentially abundant orders or genera and physiological parameters (Figure 6B; Supplementary Figure S3). Most notable were positive correlations between Verrucomicrobiales levels in the magnum and average egg weight, magnum length and oviduct length. Levels of *Blautia* in the magnum were negatively correlated with magnum length, isthmus length and total oviduct length.

## Discussion

The main result shown in this analysis is that modern Cobb breeders, which are the result of an extensive breeding program, and Legacy breeders, which have not undergone selection since 1986, have different magnum and infundibulum microbiomes. To make sure that differences in bacterial colonization are the result of genetics and not an artifact, a number of conditions were met. First, these hens were grown together from the day of hatch and kept together under the same conditions, including feed and handlers. Secondly, the analysis was performed at the age of 37 weeks. This allowed multiple cross-contamination events to occur between the hens. Last, at this age both breeds were still at peak laying performance. This ensured that they were at the same physiological state of life – well into adulthood. Consequently, as all of these possible confounders were excluded, the differences reported are likely to be due to differences in the genetics of the two breeds.

Previous work has already implied that the composition of the reproductive tract microbiome is very much affected by the gut microbiome (1). Indeed, the analysis described for both breeds analyzed here reaches the same conclusion. Here it was shown that the order Verrucomicrobiales was found to be significantly higher in the infundibulum and magnum of Cobb dams, and Lactobacillales was significantly lower in the infundibulum, mirroring the significant differences shown in the comparison of the cecum microbiota of the two breeds (13). Furthermore, Bacteroidales which was found to be significantly higher in the infundibulum of Cobb dams was also high in the cecum of Cobb dams, though not significantly so. Thus, it is possible that the differences in the reproductive tract microbiome between the Legacy and Cobb hens shown here are a result of physiological differences affecting the gut environment rather than the reproductive tract environment. However, differences in the orders Pseudomonadales, YS2 and RF32, reported in this work, were not found in previously reported cecum samples (13) and may imply additional differences also in the reproductive tract environment.

Differences in the reproductive tract microbiome may have a number of implications for hen physiology and production efficiency. First, as eggs develop along the reproductive tract, the presence of different microbiomes may affect egg production. The presence of Verrucomicrobiales which include mucin degrading *Akkermensia* bacteria in Cobb breeders is especially of interest. Notably, Verrucomicrobiales represent about 4% of the Cobb magnum while close to 0% in the Legacy breeder’s magnum, and Verrucomicrobiales correlated well with both average egg size as well as magnum and oviduct lengths. It is not known how extensive degradation of mucin in the magnum might affect albumin secretion by the magnum. Whether identified differences in reproductive tract microbiome composition are responsible for the differences in egg weight shown here is unknown. Second, the presence of microbes in the infundibulum might affect fertilization. Interestingly, Cobb breeders have high levels of Bacteriodales (17.13%) in their infundibulum compared to Legacy breeders (6.33%), while Legacy breeders have high levels of Lactobacillales (36.43%) compared to Cobb breeders (14.08%). Because Lactobacillales secrete lactate and acetate, while Bacteroidales also produce propionate, it is possible that the environment in which fertilization occurs is different in the two breeds. How do differing levels of short chain fatty acids affect fertilization or gene regulation and metabolism of the embryo are unknown. Third, the differences in microbiome composition along the reproductive tract might affect the movement of sperm up the reproductive tract. Again, of special interest is the difference in Verrucomicrobiales and their possible effect on mucin levels. Finally, it is known that the microbiota produces signals that affect the maturation and function of host systems such as the intestinal, immune and neurological systems (25–29). Thus, it can be speculated that differences in the reproductive tract microbiota shown here between Cobb and Legacy dams might contribute to the differing length of their magnum section. To conclude, the differences in the reproductive tract microbiomes of the two breeds raise many interesting questions on the effects of these differences on fertilization and egg production.

Another possible result of the differences in microbiome composition between the two breeds relate to the possible vertical transmission of gut bacteria to chicks. Previous work looking into possible vertical transmission of bacteria utilizing the egg as a vehicle for transmission showed that only a few bacterial strains, if any, were able to use this route of transmission (7). However, *Lactobacillus* strains were prime suspects in utilizing the egg for transmission. Considering that Lactobacillales were significantly higher in both the magnum and infundibulum of Legacy breeders compared to Cobb breeders, it is possible that vertical transmission of Lactobacillales is different in the two breeds.

A limitation of this work is that the reproductive tract microbiome was analyzed by 16S rRNA sequencing which is based on the presence of DNA. Importantly, it is not known if the bacteria described here, which were identified by the presence of the relevant DNA, were alive at the time of sampling. Because the reproductive tract, and especially the magnum section of the reproductive tract, secret many antibacterials, it is possible that some of the bacteria that reach the reproductive tract die in this environment. Further work analyzing the culturable reproductive tract microbiota is required to better represent this microbiome. Nevertheless, the results presented here show that at the very least the microbiome inputs of Cobb and Legacy breeders differ.

## Conclusion

In this study, we compared the composition of the reproductive tract microbiota of the modern Cobb commercial breeder line and a Legacy line which has not undergone selection for 35 years. By growing dams of the two lines under the same management protocol and in the same hen house for 37 weeks, we removed confounders and were able to identify differences in the reproductive tract bacterial community that were the result of genetic differences between the two breeds. Thus, genetics affect the composition of the reproductive tract microbiome. Finally, this work also raises a number of questions regarding the possible effect of the identified differences in reproductive tract microbiome on fertilization, egg production and vertical transmission of symbiotic bacteria.

## Data availability statement

The datasets presented in this study can be found in online repositories. The names of the repository/repositories and accession number(s) can be found at: https://www.ncbi.nlm.nih.gov/, PRJNA1074539.

## Ethics statement

The animal study was approved by Hebrew University of Jerusalem’s Ethics committee. The study was conducted in accordance with the local legislation and institutional requirements.

## Author contributions

NS: Writing – review & editing, Writing – original draft, Visualization, Methodology, Investigation, Formal analysis, Data curation, Conceptualization. YS: Writing – review & editing, Investigation. BP: Writing – review & editing, Investigation. NR: Writing – review & editing, Investigation. OO: Writing – review & editing, Investigation. SD: Writing – review & editing, Resources, Investigation. EM: Writing – review & editing, Writing – original draft, Supervision, Resources, Project administration, Investigation, Funding acquisition, Conceptualization.

## Footnotes

^1^ www.graphpad.com
