# Supplementary material for: Comparative analysis of reproductive tract microbiomes in modern and slower-growing broiler breeder lines
Source: Front Vet Sci. 2024 Apr 10;11:1386410. doi: 10.3389/fvets.2024.1386410 (PMC11039882; doi:10.3389/fvets.2024.1386410)
Supplement: Supplementary file 1 [file Table_1.DOCX]

Supplementary Table S1: Feed nutritional composition

| Age (weeks) | 0-2 | 2-4 | 5-16 | 17-22 | 23-40 |
| --- | --- | --- | --- | --- | --- |
| Protein (%) | 19 | 18 | 14.5 | 15 | 15 |
| Calcium (%) | 1 | 1 | 1.1 | 1.4 | 2.9 |
| Phosphorus (%) | 0.73 | 0.7 | 0.63 | 0.66 | 0.62 |
| Fat (%) | 3.5 | 3.5 | 3 | 3 | 3.5 |
| Fiber (%) | 3.5 | 3.5 | 4.5 | 3.5 | 3.5 |
| Ash (%) | 5.5 | 5.5 | 5.5 | 7.5 | 10 |
| Salt (%) | 0.35 | 0.335 | 0.35 | 0.35 | 0.35 |
| Manganese (g) | 100 | 100 | 90 | 110 | 110 |
| Moisture (%) | 13 | 13 | 13 | 13 | 13 |
| Linoleic acid (%) | 1.2 | 1.25 | 1.1 | 1.3 | 1.3 |
| Metabolizable energy (kcal/kg) | 2800 | 2750 | 2650 | 2750 | 2750 |
