# Supplementary material for: Comparative analysis of reproductive tract microbiomes in modern and slower-growing broiler breeder lines
Source: Front Vet Sci. 2024 Apr 10;11:1386410. doi: 10.3389/fvets.2024.1386410 (PMC11039882; doi:10.3389/fvets.2024.1386410)
Supplement: Supplementary file 2 [file Data_Sheet_1.PDF]

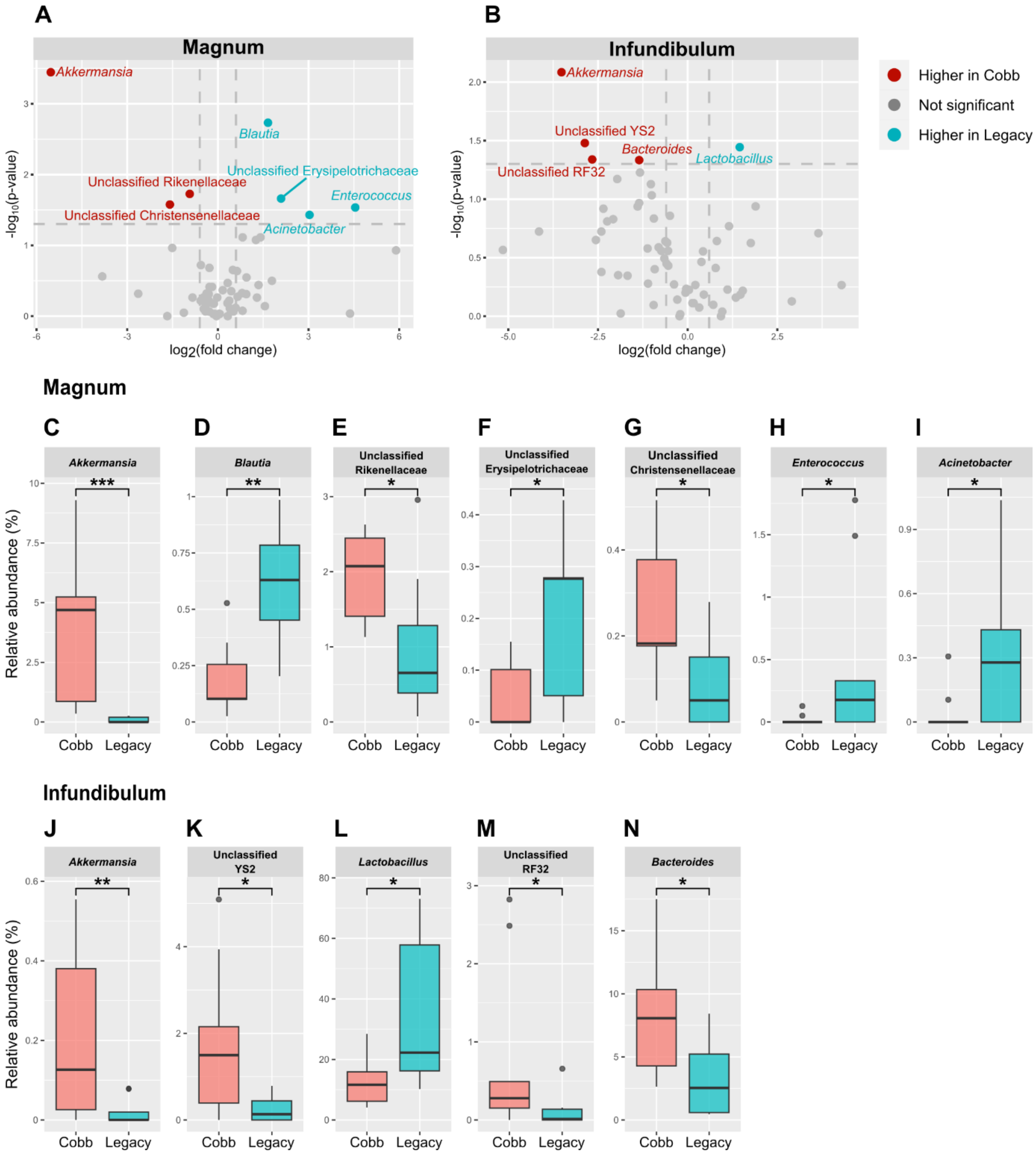

Figure S1: Differentially abundant genera between Cobb and Legacy dams in magnum and infundibulum samples. A, B) Volcano plot of genera with average relative abundance > 0.1% in the magnum (A) and infundibulum (B). Genera with relative abundance significantly higher in Cobb dams are marked red, genera with relative abundance significantly higher in Legacy dams are marked blue, and genera not significantly different are marked gray. C, D) Relative abundance of genera significantly different between Cobb and Legacy dams in the magnum (C-I) and infundibulum (J-N). * Mann-Whitney $p < 0.05$, ** $p < 0.01$, *** $p < 0.001$.

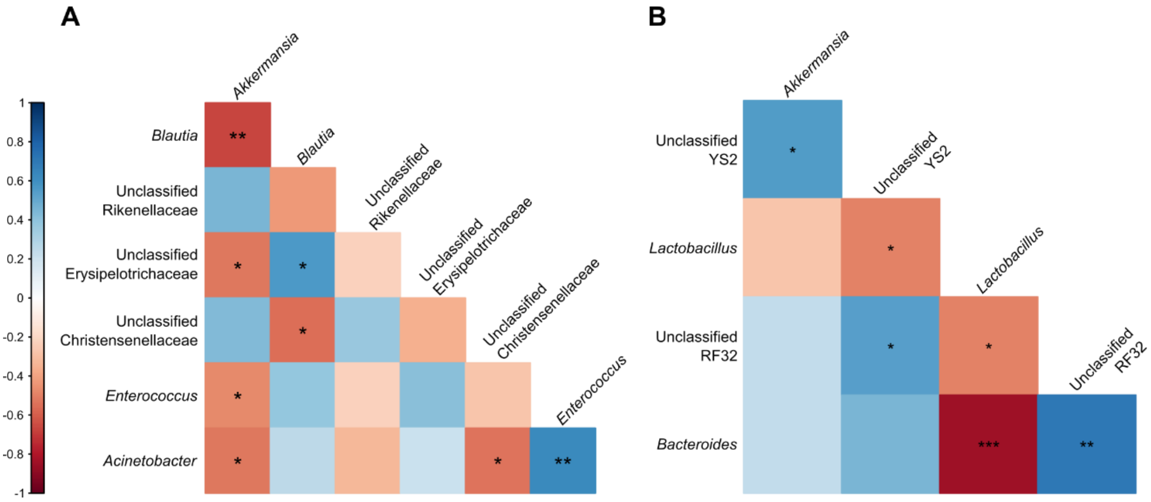

<u>Figure S2</u>: Associations between differentially abundant genera. Spearman correlation was used to assess genera associations in the magnum (A) and in the infundibulum (B). Spearman r values are represented by a color scale, and correlation significance is denoted with asterisks. * $p < 0.05$, ** $p < 0.01$, *** $p < 0.001$.

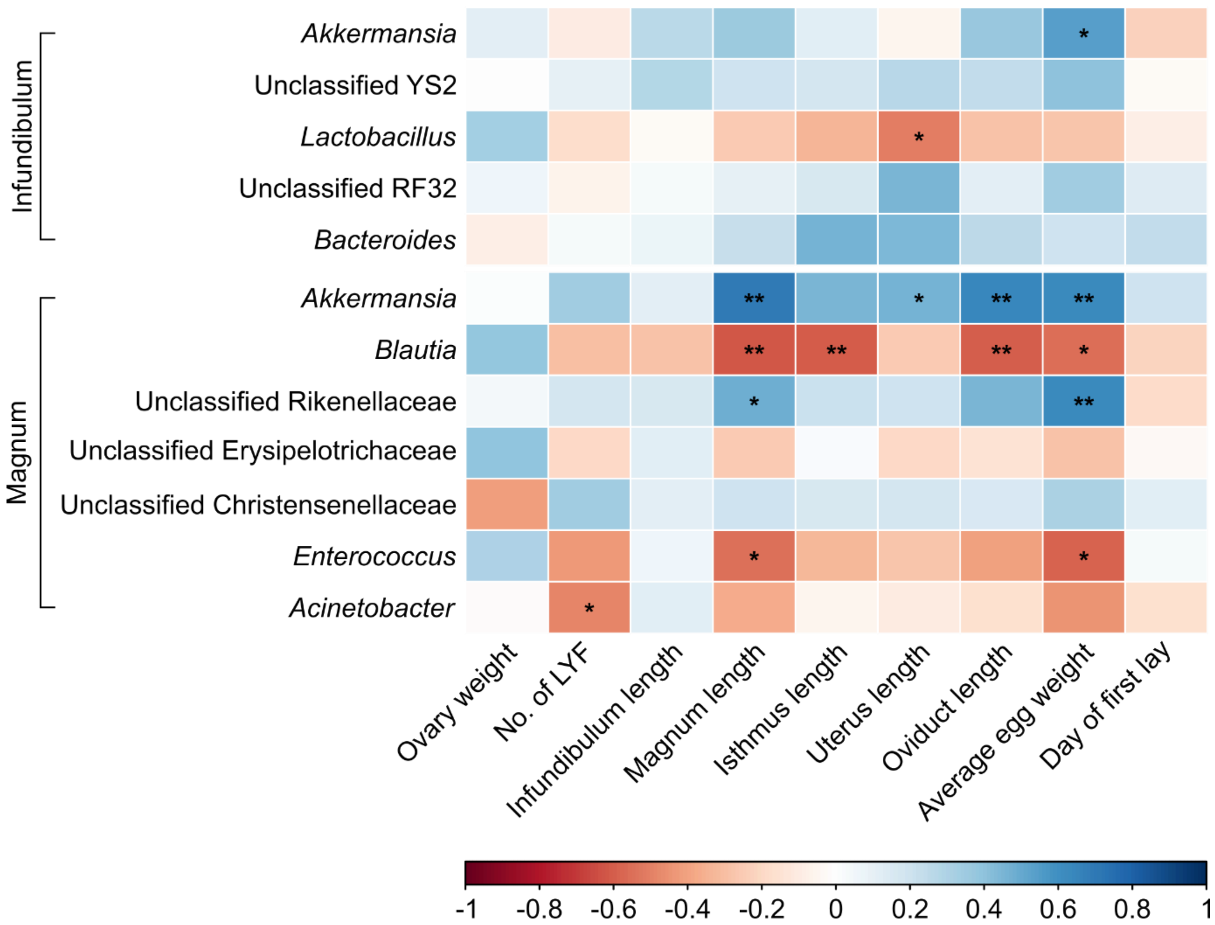

Figure S3: Correlations between differentially abundant genera and physiological parameters. Spearman correlation was used to assess associations. Spearman r values are represented by a color scale, and correlation significance is denoted with asterisks. * $p < 0.05$, ** $p < 0.01$, *** $p < 0.001$.
